# Predictors associated with neurological recovery after anterior decompression with fusion for degenerative cervical myelopathy

**DOI:** 10.1186/s12893-021-01147-w

**Published:** 2021-03-19

**Authors:** Hiroyuki Inose, Takashi Hirai, Toshitaka Yoshii, Atsushi Kimura, Katsushi Takeshita, Hirokazu Inoue, Asato Maekawa, Kenji Endo, Takeo Furuya, Akira Nakamura, Kanji Mori, Shunsuke Kanbara, Shiro Imagama, Shoji Seki, Shunji Matsunaga, Kunihiko Takahashi, Atsushi Okawa

**Affiliations:** 1 Department of Orthopaedic and Trauma Research, Tokyo Medical and Dental University, 1-5-45 Yushima, Bunkyo-ku, Tokyo, 113-8519 Japan; 2 Department of Orthopaedic Surgery, Tokyo Medical and Dental University, Tokyo, Japan; 3 Department of Orthopaedics, Jichi Medical University, Shimotsuke, Japan; 4 Department of Orthopaedic Surgery, Tokyo Medical University, Tokyo, Japan; 5 Department of Orthopedic Surgery, Chiba University Graduate School of Medicine, Chiba, Japan; 6 Department of Orthopaedic Surgery, Shiga University of Medical Science, Otsu, Japan; 7 Department of Orthopedic Surgery, Nagoya University Graduate School of Medicine, Nagoya, Japan; 8 Department of Orthopedic Surgery, Faculty of Medicine, University of Toyama, Toyama, Japan; 9 Department of Orthopedic Surgery, Imakiire General Hospital, Kagoshima, Japan; 10 Department of Biostatistics, M&D Data Science Center, Tokyo Medical and Dental University, Tokyo, Japan

**Keywords:** Spinal Stenosis, Spondylosis, Ossification of Posterior Longitudinal Ligament, Neck Pain, Spinal Fusion

## Abstract

**Background:**

Anterior decompression with fusion (ADF) has often been performed for degenerative cervical myelopathy (DCM) in patients with poor cervical spine alignment and/or anterior cord compression. We aimed to identify clinical and radiological predictors associated with neurological recovery after ADF.

**Methods:**

This post-hoc analysis from a prospective multicenter study included patients who were scheduled for ADF for DCM. The patients who received other surgeries (laminoplasty, posterior decompression and fusion) were excluded. The associations between baseline clinical and radiographic variables (age, sex, body mass index, etiology, cervical lordosis, range of motion, C7 slope, C2-7 sagittal vertical axis [SVA], thoracic kyphosis [TK], lumbar lordosis, sacral slope, SVA, pelvic tilt, T1 pelvic angle [TPA], the Japanese Orthopedic Association score for the assessment of cervical myelopathy [C-JOA], European Quality of Life Five Dimensions Scale [EQ-5D], Neck Disability Index [NDI], Physical Component Summary of the SF-36 [PCS], and Mental Component Summary of the SF-36) and the recovery rates as the outcome variables were investigated in the univariate regression analysis. Then, the independent predictors for increased recovery rates were evaluated using a stepwise multiple regression analysis.

**Results:**

In total, 37 patients completed the 1 year follow-up. The recovery rate was significantly correlated with SVA (p = 0.001) and TPA (p = 0.03). Univariate regression analyses showed that age (Regression coefficient = − 0.92, *p* = 0.049), SVA (Regression coefficient  = − 0.57, *p* = 0.004) and PCS (Regression coefficient = 0.80, *p* = 0.03) score were significantly associated with recovery rate. Then, a stepwise multiple regression analysis identified the independent predictors of recovery rate after ADF as TK (*p* = 0.01), PCS (*p* = 0.03), and SVA (*p* = 0.03). According to this prediction model, the following equation was obtained: recovery rate = − 8.26 + 1.17 × (TK) − 0.45 × (SVA) + 0.85 × (PCS) (*p* = 0.002, *R*^2^ = 0.44).

**Conclusion:**

Patients with lower TK, lower PCS score, and higher SVA were more likely to have poor neurological recovery after ADF. Therefore, patients with DCM and these predictors who undergo ADF should be warned about poor recovery and be required to provide adequate informed consent.

## Background

With age, degeneration of the cervical spine progresses [1]. Accordingly, with the advent and progression of an aging society, the number of patients with degenerative cervical myelopathy (DCM) will increase. Surgery is considered to be a treatment for advanced DCM that is resistant to conservative therapy and interferes with daily life [2]. There are two types of surgery for DCM: anterior and posterior approaches, and their surgical outcomes are generally comparable [3, 4]. Anterior decompression with fusion (ADF) has often been performed for patients with poor cervical spine alignment and/or anterior cord compression [5]. Although the results of ADF are usually satisfactory, sometimes they are not. At present, unfortunately, it has been difficult to preoperatively predict the extent of neurological improvement a patient will experience after ADF.

To date, research regarding preoperative predictors for the success of ADF has been limited. A prospective randomized study showed that preoperative predictive factors of good outcome 10–13 years after ADF included initial high neck-related pain intensity, nonsmoking status at the time of surgery, and male sex [6]. A retrospective study showed that advanced age, longer duration of symptoms, and bigger kyphotic angle at final follow-up were associated with poor outcome in DCM patients after anterior surgery [7]. Regarding radiographic parameters, the presence of intramedullary signal changes on T2-weighted sequences on MRI in patients with DCM suggests a poor prognosis [8, 9]. However, as far as we are aware, there is limited information regarding the analysis of how whole spinal parameters play a role in surgical outcomes after ADF.

The aim of this study was to identify clinical and radiological predictors, including whole spinal radiographic parameters, associated with neurological recovery after ADF.

## Methods

### Study population

This study was a secondary analysis of a previous prospective multicenter study that investigated the characteristics of patients with DCM and their surgical outcomes [10]. Briefly, the original study, initiated by the Japanese Organization of the Study for Ossification of the Spinal Ligament, prospectively recruited patients with cervical myelopathy who were scheduled for surgical treatment at eight participating institutes (Tokyo Medical and Dental University, Jichi Medical University, Tokyo Medical University, Chiba University, Shiga University of Medical Science, Nagoya University, University of Toyama, and Imakiire General Hospital) from October 2016 through December 2017 [10]. Institutional review board approval was obtained before initiation of the study. At the time of enrollment, written informed consent was obtained from all participants. Demographic data, including age, sex, body mass index, and etiology of myelopathy were collected. The exclusion criteria were comorbidities impairing physical functions (e.g., cerebral infarction, cerebral palsy, or severe rheumatoid arthritis), bedridden status or full dependence on a wheelchair before surgery due to severe cervical myelopathy, and difficulty completing a questionnaire because of cognitive impairment.

**Fig. 1:**
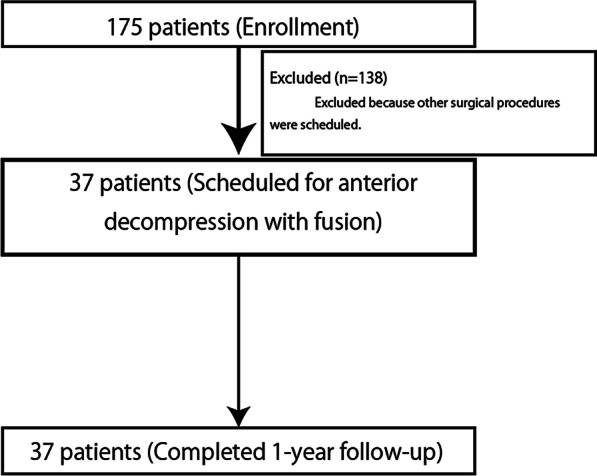
Participant flow through the study. During the study period, 175 patients were enrolled in the original study, and 138 patients were excluded because other surgical procedures were scheduled

The current study included patients with DCM who had undergone ADF. Accordingly, patients who received other surgeries (laminoplasty, posterior decompression and fusion) were excluded (Fig. 1). This study was approved by the institutional review board at the Tokyo Medical and Dental University and was compliant with the Declaration of Helsinki.

### Radiologic findings

As radiological parameters, we measured cervical lordosis (CL), C2-7 range of motion (ROM), C7 slope, C2-7 sagittal vertical axis (SVA), thoracic kyphosis (TK), lumbar lordosis (LL), sacral slope (SS), SVA, pelvic tilt (PT), and t1 pelvic angle (TPA).

CL was defined by the Cobb angle between C2 and C7 on a lateral radiograph in the neutral position. C2-7 ROM was measured on flexion–extension lateral radiographs. The C7 slope was calculated by measuring the angle formed by the horizontal line to the C7 vertebra and the line parallel to the superior endplate of the C7 vertebra [11]. TK was defined by the Cobb angle between the superior endplate and the inferior endplate of T1-T12 [12]. The C2-7 SVA was defined as the sagittal distance between a plumb line dropped from the center of C2 and the posterior superior corner of C7 [13]. LL was defined as the angle between the superior endplate of L1 to the inferior endplate of L5 [14]. SS was measured between the tangent line to the superior endplate of S1 and the horizontal plane [15]. SVA was defined as the sagittal distance between the C7 plumb line and the vertical line through the posterosuperior corner of the S1 endplate on standing whole-spine lateral radiographs [16]. PT was measured as the angle between the vertical reference line from the center of the femoral head and the line from the center of the femoral head to the midpoint of the sacral endplate [17]. TPA was defined as the angle between the line from the femoral head axis to the centroid of T1 and the line from the femoral head axis to the middle of the S1 endplate [18].

### Outcome measures

Outcomes were assessed before surgery and 1 year after surgery using the Japanese Orthopedic Association score for the assessment of cervical myelopathy (C-JOA score, which ranges from 0 to 17, with higher scores indicating better neurological function) [19], the European Quality of Life Five Dimensions Scale (EQ-5D, which ranges from -0.111 to 1, with higher scores indicating better quality of life) [20], the Neck Disability Index (NDI) score (which ranges from 0 to 100, with higher percentages indicating more severe disability) [21], and the SF-36 Physical Component Summary (PCS) and Mental Component Summary (MCS) (which range from 0 to 100, with higher scores indicating better health and functioning) [22]. The recovery rate, based on the C-JOA score, was calculated according to Hirabayashi’s method using the following formula: recovery rate (%) = (postoperative C-JOA score − preoperative C-JOA score) × 100 / (17 − preoperative C-JOA score) [23].

### Statistical analysis

We performed a paired two-tailed t-test for normally distributed data or a Wilcoxon signed-rank test for skewed distributed data to identify differences in scores before surgery and 1 year post-surgery after assessing normality with the Shapiro–Wilk test. Spearman correlation coefficients were used to evaluate the relationships between the recovery rate and the preoperative radiographic factors, and between TK and other radiographic factors. A score of 0.0–0.2 represents “very weak or no correlation”; 0.2–0.4, “weak correlation”; 0.4–0.6, “moderate correlation”; 0.6–0.8, “strong correlation”; and 0.8–1.0, “very strong correlation. The associations between baseline clinical and radiographic variables with recovery rate were investigated with a multiple linear regression model. First, predictors associated with the dependent variable at a *p*-value < 0.25 in univariate regression analyses were carried forward to the second step of the analysis [24]. Second, the stepwise multiple regression analysis was conducted by using the recovery rate (dependent variable) and the remaining predictors (candidates for independent variables based on the results of univariate regression analysis: age, TK, SVA, TPA, NDI, and PCS) to determine the best sets of predictors.

Predictors with a *p*-value > 0.05 were removed. For all statistical analyses, JMP version 12 (SAS Institute, Cary, NC, USA) was used, and a p-value of < 0.05 was considered statistically significant.

## Results

### Patient demographics and surgical outcomes

A total of 37 patients with 1 year of postoperative follow-up were included in this study. The baseline characteristics of the patients are shown in Table 1. The mean age of the patients was 60.3 years. The average cervical lordosis was 9.5° before surgery.

**Table 1:** Demographic data of patients

Number of cases	37
Age (year)	60.3 ± 11.3
Female sex [no. (%)]	14 (38)
BMI	25.6 ± 4.6
OPLL [no.(%)]	15 (41)
CL (degree)	9.5 ± 12.3
ROM (degree) (n = 36)	32.3 ± 17.9
C7 slope (degree)	22.3 ± 10.7
C2-7 SVA (mm)	20.0 ± 13.8
TK (degree) (n = 35)	34.0 ± 12.8
LL (degree) (n = 35)	35.3 ± 12.3
SS (degree) (n = 35)	29.1 ± 7.6
SVA (mm) (n = 35)	23.4 ± 27.0
PT (degree) (n = 34)	18.8 ± 6.2
TPA (degree) (n = 34)	15.1 ± 6.0

*BMI* body mass index, *OPLL* ossification of posterior longitudinal ligament, *CL* cervical lordosis, *ROM* range of motion, *SVA* sagittal vertical axis, *TK* thoracic kyphosis, *LL* Lumbar lordosis, *SS* sacral slope, *PT* pelvic tilt, *TPA* t1 pelvic angle

Data are given as mean ± SD

Table 2 shows the surgical outcomes. The mean recovery rate was 45.7%. While C-JOA, EQ-5D, NDI, and PCS scores were improved postoperatively, there was no significant difference between the preoperative and 1 year postoperative MCS scores (Table 2).

**Table 2:** Surgical outcomes

Characteristic	pre	1 year after surgery	p
C-JOA	11.0 ± 2.3	13.6 ± 2.5	< 0.001*^a^
Recovery rate	45.7 ± 31.7		
EQ-5D	0.55 ± 0.17 (n = 33)	0.68 ± 0.18 (n = 34)	< 0.001*^a^
NDI	42.8 ± 19.5 (n = 32)	28.5 ± 19.3 (n = 34)	< 0.001*^b^
PCS	26.0 ± 15.9 (n = 32)	35.8 ± 16.4 (n = 34)	< 0.001*^b^
MCS	47.8 ± 9.2 (n = 32)	50.9 ± 9.1 (n = 34)	0.08^b^

A Wilcoxon signed-rank test^a^ or a paired t-test^b^ was used to compare the preoperative and postoperative values

*C-JOA* Japanese Orthopedic Association score for the assessment of cervical myelopathy, *EQ-5D* European Quality of Life-5 Dimensions, *NDI* neck disability index, *PCS* Physical component summary of SF36, *MCS* Mental component summary of SF36

*p < 0.05

Data are given as mean ± SD

### Correlations between recovery rate and preoperative radiographic factors

We then investigated whether the recovery rate correlated with the preoperative factors (Table 3). The results showed that the recovery rate significantly negatively correlated with the preoperative SVA (*ρ* = − 0.52, *p* = 0.001) and TPA (*ρ* = − 0.38, *p* = 0.03); however, no correlations were observed between the recovery rate and other radiographic parameters. Based on the Spearman correlation coefficients, SVA had a moderate correlation with the recovery rate, and TPA had a weak correlation with the recovery rate.

**Table 3:** Correlations between recovery rate and preoperative radiographic parameters

	*ρ*	*p*-value
Recovery rate versus		
CL	− 0.05	0.78
ROM	− 0.11	0.50
C7 slope	− 0.05	0.77
C2-7 SVA	− 0.02	0.90
TK	0.24	0.17
LL	0.08	0.66
SS	0.13	0.47
SVA	− 0.52	0.001*
PT	− 0.13	0.47
TPA	− 0.38	0.03*

Spearman correlation coefficient was used to evaluate the relationships between the recovery rate and the preoperative radiographic factors

*CL* cervical lordosis, *ROM* range of motion, *SVA* sagittal vertical axis, *TK* thoracic kyphosis, *LL* Lumbar lordosis, *SS* sacral slope, *PT* pelvic tilt, *TPA* t1 pelvic angle

*p < 0.05

### Independent predictors of recovery rate after ADF

The association between the baseline variables and the recovery rate was investigated in a univariate regression model (Table 4). The univariate regression analysis showed that age, SVA, and PCS score were significantly associated with the recovery rate after ADF (*p* = 0.049, 0.004 and 0.03).

**Table 4:** Univariate regression analysis. Association of baseline variables with recovery rate

Characteristic	Regression coefficient	95% CI	*P*
Age (year)	− 0.92	− 1.83–− 0.004	0.049*
Female sex [no. (%)]	6.08	− 4.78–16.95	0.26
BMI	0.97	− 1.38–3.32	0.41
OPLL	4.66	− 6.16–15.47	0.39
CL	− 0.47	− 1.33–0.40	0.28
ROM	− 0.26	− 0.88–0.35	0.39
C7 slope	− 0.17	− 1.19–0.84	0.73
C2-7 SVA	− 0.002	− 0.79–0.79	0.996
TK	0.51	− 0.37–1.38	0.245
LL	0.004	− 0.93–0.94	0.99
SS	0.26	− 1.24–1.76	0.73
SVA	− 0.57	− 0.94–− 0.20	0.004*
PT	− 0.10	− 1.92–1.70	0.90
TPA	− 1.43	− 3.24–0.39	0.12
C-JOA	2.18	− 2.45–6.82	0.35
EQ-5D	34.46	− 33.49–102.40	0.31
NDI	− 0.40	− 1.00–0.20	0.18
PCS	0.80	0.10–1.49	0.03*
MCS	− 0.30	− 1.61–1.00	0.64

The associations between baseline variables with recovery rate were investigated with a univariate linear regression model

*CI* confidence interval, *BMI* body mass index, *OPLL* ossification of posterior longitudinal ligament, *CL* cervical lordosis, *ROM* range of motion, *SVA* sagittal vertical axis, *TK* thoracic kyphosis, *LL* Lumbar lordosis, *SS* sacral slope, *PT* pelvic tilt, *TPA* t1 pelvic angle, *C-JOA* Japanese Orthopedic Association score for the assessment of cervical myelopathy, *NDI* neck disability index, *EQ-5D* European Quality of Life-5 Dimensions, *PCS* Physical component summary of SF36, *MCS* Mental component summary of SF36

*p < 0.05

**Fig. 2:**
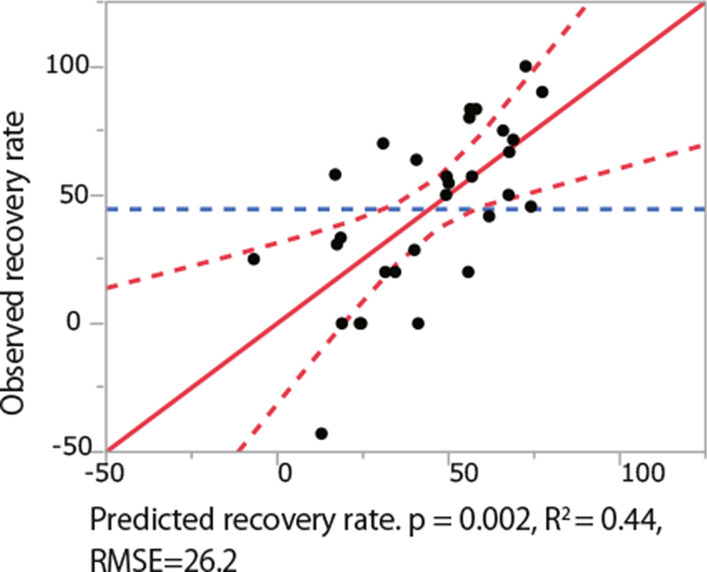
Observed versus predicted plots of the multiple linear regression model for recovery rate

Then, the independent predictors for recovery rate were investigated using a stepwise multiple regression analysis. Based on the univariate analysis, the dependent variable was defined as the recovery rate, and the candidate independent variables were age, TK, SVA, TPA, NDI, and PCS. As a result, the independent baseline predictors were identified as TK (Regression coefficient = 1.17, *p* = 0.01), PCS (Regression coefficient = 0.85, *p* = 0.03), and SVA (Regression coefficient = − 0.45, *p* = 0.03) (Tables 5 and 6. Using the independent predictors obtained in the stepwise regression analysis, the following equation was obtained: recovery rate = − 8.26 + 1.17 × (TK) − 0.45 × (SVA) + 0.85 × (SF-36′s PCS) (Fig. 2).

**Table 5:** Multiple regression analysis: independent predictors of recovery rate

Factor	Regression coefficient	95% CI	*P*
TK	1.17	0.27–2.06	0.01*
PCS	0.85	0.11–1.58	0.03*
SVA	− 0.45	− 0.85–− 0.05	0.03*

The associations between baseline variables with recovery rate were investigated with a multiple linear regression model

*CI* confidence interval, *TK* thoracic kyphosis, *PCS* Physical component summary of SF36, *SVA* sagittal vertical axis

*p < 0.05

**Table 6:** Correlations between thoracic kyphosis and other radiographic parameters

	*ρ*	*p*-value
Thoracic kyphosis versus		
CL	0.27	0.12
ROM	− 0.21	0.24
C7 slope	0.66	< 0.001*
C2-7 SVA	0.40	0.02*
LL	0.35	0.04*
SS	0.20	0.24
SVA	− 0.002	0.99
PT	0.05	0.76
TPA	0.08	0.64

Spearman correlation coefficient was used to evaluate the relationships between the thoracic kyphosis and other radiographic factors

*CL* cervical lordosis, *ROM* range of motion, *SVA* sagittal vertical axis, *LL* lumbar lordosis, *SS* sacral slope, *PT* pelvic tilt, *TPA* t1 pelvic angle

*p < 0.05

Lastly, we examined the correlation between TK and other radiographic parameters. The results showed that TK correlated with the C2-7SVA (*ρ* = 0.40, *p* = 0.02), C7 slope (*ρ* = 0.66, *p* < 0.001), and LL (*ρ* = 0.35, *p* = 0.04). Based on the Spearman correlation coefficients, C2-7 SVA and LL had weak correlations with TK, and C7 slope had a strong correlation with TK.

## Discussion

This study investigated the predictors of recovery rate after ADF. C-JOA, EQ-5D, NDI, and PCS scores were improved postoperatively. The recovery rate was significantly correlated with the SVA and TPA. Univariate regression analyses showed that the age, SVA, and PCS scores were significantly associated with the recovery rate. Lastly, stepwise multiple regression analysis showed that the independent predictors of recovery rate after ADF were identified as TK, PCS, and SVA. To the best of our knowledge, this study is the first to investigate the predictive value of global spinal parameters and PCS score for recovery rate after ADF.

The clinical outcomes of ADF were limited by various factors, such as duration of symptoms, age, BMI, and preoperative MRI spinal cord signal changes [2, 25]. Regarding the association between radiographic parameters and surgical outcomes after ADF, in a retrospective study comparing ADF results between young-old patients and middle-old patients, there was no significant difference in cervical lordosis and C2-7SVA between the two groups and no significant difference in clinical outcomes [26]. Another retrospective study showed that low postoperative cervical spine alignment change was a risk factor for poor recovery after ADF [27]. In this study, we found that the SVA and TPA were significantly negatively correlated with the recovery rate after ADF. This is the first time, to the best of our knowledge, that these correlations have been identified. The TPA is a radiographic measure of sagittal spinal alignment, and a low TPA indicates good thoracic-lumbar alignment [28]. Accordingly, the surgeon should consider the TPA when performing ADF on patients with DCM. One other advantage of the TPA is that it is less affected by patient posture, as the TPA can also be measured in a seated position [28]. Therefore, in the case of patients who have difficulty standing due to severe myelopathy, measuring the TPA may be substituted for the SVA to predict neurological recovery after ADF. We also found that CL was not correlated with the recovery rate. This result confirmed the notion that even in patients with poor cervical spine alignment, ADF can be expected to produce good neurological recovery.

We also found that the independent radiographic predictors of recovery rate after ADF were identified as TK and SVA. These results indicate that patients with higher TK and lower SVA were more likely to improve their C-JOA scores after ADF. A recently established concept is that good SVA can be predicted by pelvic incidence, LL, and TK [29]. In general, as thoracic kyphosis increases, lumbar lordosis increases to maintain the C7 in the correct position. Conversely, as lumbar lordosis decreases, thoracic kyphosis decreases [30]. Indeed, in the present study, TK showed a weak positive correlation with LL, even though it was not significantly correlated with SVA. Collectively, the results of this study may be interpreted to mean that good ADF results can be obtained when there is better sagittal balance with low SVA, and when TK and LL are not flat but are in a physiological curved balance. However, it is important to note that when the compensation mechanism for spinal kyphosis is surpassed, spinal kyphosis continues to progress. In such a condition, even if lumbar lordosis decreases, thoracic kyphosis may increase.

We also found that the independent predictor of recovery rate after ADF was identified as PCS score. A cohort study that investigated the surgical results of cervical spondylotic myelopathy showed that the recovery rate improvements correlated with the physical component domains of SF-36 [31], although both anterior and posterior surgery scores were included for the analysis. Collectively, when performing ADF, it may be important to have a thorough understanding of the patient's physical functioning prior to surgery to accurately predict the postoperative neurological recovery.

There are some limitations in this study. First, although our study is based on prospectively collected data, the primary limitation of this study is the retrospective design. Second, sample size may have been small. Although there is no consensus on the appropriate sample size for the multiple regression analysis, in some studies, 10 events per variable is considered reasonable in a regression analysis [32, 33]. Based on this idea, the sample size of this study was 37, which is considered sufficient since there are more than 30 patients (10 patients per variable, for 3 variables in the multiple regression model in this study). Further prospective studies are needed to address these limitations and validate the results of this study.

## Conclusions

We found that the recovery rate following ADF was negatively correlated with the preoperative SVA and TPA. A preoperatively higher SVA, lower TK, and lower PCS score were independent predictors for poor recovery after ADF. Therefore, patients who undergo ADF with these predictors might be cautioned about poor recovery and be required to provide adequate informed consent.

## Data Availability

The datasets generated and/or analyzed during the current study are not publicly available due to conditions of ethical approval but are available from the corresponding author on reasonable request.

## Abbreviations

ADF: Anterior decompression with fusion
DCM: Degenerative cervical myelopathy
PCS: Physical Component Summary of the SF-36
SVA: Sagittal vertical axis
TK: Thoracic kyphosis
CL: Cervical lordosis
TPA: T1 pelvic angle
C-JOA score: Japanese Orthopedic Association score for the assessment of cervical myelopathy
EQ-5D: European Quality of Life Five Dimensions Scale
NDI: Neck Disability Index
MCS: Mental Component Summary of the SF-36
